# An Introduction to Bacterial Biofilms and Their Proteases, and Their Roles in Host Infection and Immune Evasion

**DOI:** 10.3390/biom12020306

**Published:** 2022-02-14

**Authors:** Juan Sebastián Ramírez-Larrota, Ulrich Eckhard

**Affiliations:** Department of Structural Biology, Molecular Biology Institute of Barcelona, CSIC, Barcelona Science Park, Baldiri Reixac, 15-21, 08028 Barcelona, Spain; jrlcri@ibmb.csic.es

**Keywords:** proteases, virulence factors, protein secretion, extracellular polymeric substances, biofilm formation and remodeling, extracellular matrix degradation, pathogenicity

## Abstract

Bacterial biofilms represent multicellular communities embedded in a matrix of extracellular polymeric substances, conveying increased resistance against environmental stress factors but also antibiotics. They are shaped by secreted enzymes such as proteases, which can aid pathogenicity by degrading host proteins of the connective tissue or the immune system. Importantly, both secreted proteases and the capability of biofilm formation are considered key virulence factors. In this review, we focus on the basic aspects of proteolysis and protein secretion, and highlight various secreted bacterial proteases involved in biofilm establishment and dispersal, and how they aid bacteria in immune evasion by degrading immunoglobulins and components of the complement system. Thus, secreted proteases represent not only prominent antimicrobial targets but also enzymes that can be used for dedicated applications in biotechnology and biomedicine, including their use as laundry detergents, in mass spectrometry for the glycoprofiling of antibodies, and the desensitization of donor organs intended for positive crossmatch patients.

## 1. The Complexity of Bacterial Biofilms

Bacteria are single-celled microorganisms that are commonly associated with being independent, free-living organisms. However, they frequently adhere to surfaces to create multi-cellular aggregates across species that interact with one another [1]. Such bacterial communities are called biofilms, and they are embedded in a self-produced extracellular polymeric substance (EPS), which not only confers increased tolerance to environmental stresses, but, as in the case of many pathogenic bacteria, also resistance against antibiotics and the host immune system. Furthermore, biofilms enhance nutrient capitalization, metabolite exchange, horizontal gene transfer, and cellular communication [2]. Importantly, because of their increased pathogenicity and resistance to therapy compared to free-living bacteria, the tendency and capacity to establish biofilms are considered as key virulence factors for a wide range of microorganisms, and the formation of biofilms on medical devices and implants, along with antibiotic resistance, poses one of the major challenges in medicine [3,4].

**Figure 1:**
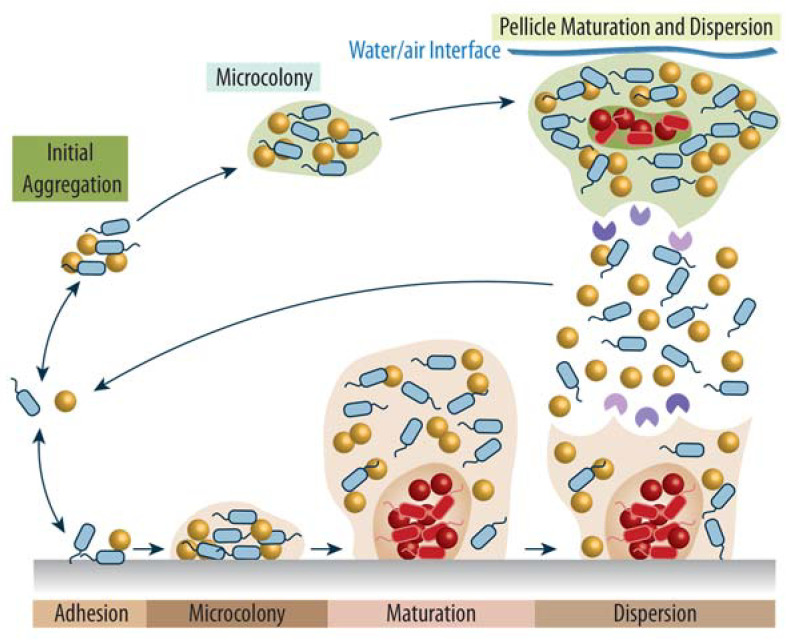
Stages of biofilm development. While the initial adhesion or aggregation of one or more planktonic bacterial species is reversible, the secretion of adhesins and different matrix biopolymers stimulates microcolony development and results in cell proliferation within the EPS. During biofilm maturation, heterogenous zones with diverse transcriptomic, proteomic, and metabolic profiles emerge, including areas with dormant bacteria and subpopulations that are lysed to provide extracellular DNA (eDNA) for the EPS. Finally, biofilms are dispersed either passively due to external forces or actively by the secretion of hydrolytic enzymes that target the EPS components, resulting in the release of planktonic bacteria or smaller cell aggregates and restarting the biofilm life cycle at new colonization sites. Exemplary bacteria are depicted as polar flagellated rods and as non-flagellated cocci in blue and in dark yellow, respectively, with their dormant counterparts in dark red. The EPS of the pellicle (top) and the surface-associated biofilm (bottom) are shown in light green and light brown, respectively, to indicate differences in chemical composition, and regions containing dormant bacteria are highlighted in a darker shade of the same hue. Purple Pacman shapes represent various secreted proteases involved in biofilm remodeling and dispersion. Figure adapted from [5,6].

Depending on the respective environment, three distinct types of biofilm growth can be identified: while pellicles (floating biofilms) are observed at air–water interfaces, colonies or submerged biofilms form on solid surfaces when interfacing with air or water, respectively (Figure 1). Despite variances in the adhesive fibers, proteins, nucleic acids, and exopolysaccharides that embed the respective biofilm, the general stages of biofilm development are remarkably similar across most bacteria, and follow four general steps: (i) reversible attachment to a surface or inaugural aggregation in solution; (ii) microcolony formation within a common EPS; (iii) biofilm growth and maturation; and (iv) dispersion and release of planktonic cells to colonize new sites [1].

In the first step, mainly driven by flagella, pili, and other adhesion proteins, planktonic bacteria arrive and reversibly bind to a surface, or, as for pellicles, form initial small cell aggregates in solution [7]. Notably, proteolytically active flagella were recently discovered in several Gram-negative and Gram-positive bacteria [8,9], and are suggested to play a critical role in biofilm remodeling in these species. The next step is marked by the secretion of high-molecular weight biopolymers, the EPS, thereby establishing the functional and structural integrity of the biofilm through a shared extracellular matrix and allowing irreversible attachment in surface-associated biofilms [1]. This typically goes hand in hand with a loss of motility, but crucially not necessarily with a loss of flagella [10,11]. At the same time, biofilm matrix polysaccharides such as Pel and Psl in *Pseudomonas aeruginosa* are increasingly produced and secreted [12], strengthening the cell–cell and cell–surface interactions. Such formed microcolonies display considerable sessile growth and cell–cell communication such as quorum sensing, allowing the individual bacteria to coordinate and carry out cooperative activities such as biofilm maturation, dispersion, and virulence [13,14]. As the biofilm matures and differentiates, phenotypically heterogeneous micro-environments and an organized 3D architecture emerge, characterized by complex internal water channels and pores that allow the distribution and transport of oxygen, nutrients, and signaling molecules necessary for concerted growth while also removing waste products and dead cells. Thus, mature biofilms exhibit multicellular-like behavior [15]. The final stage of biofilm development is distinguished by the active (i.e., initiated by the bacteria) or passive (i.e., mediated by external forces) dispersal of the mature biofilm and the release of single planktonic cells, clumps, or pieces of biofilm into the environment to colonize new sites and begin a new cycle of biofilm formation. Importantly, numerous extracellular hydrolases, including saccharolytic enzymes and especially proteases, are produced during the active cell release from the EPS to destabilize and break down the EPS matrix [16,17,18]. Importantly, each of the biofilm stages is associated with specific transcriptomic, proteomic, and metabolomic profiles, reflecting bacterial adaptation to the respective situation. Likewise, distinct profiles are observed in the differentiated regions of the mature biofilm [19,20].

The key components of any EPS are highly hydrated biosynthesized polymers such as polysaccharides, proteins, and DNA at various ratios depending on the bacterial species or even strain, but also strongly depending on the actual type of biofilm formed, the biofilm morphotype [17,21]. Thus, the EPS is characterized by remarkable diversity that also allows the biofilm to adapt to different environments depending on environmental signals and quorum sensing. The EPS matrix provides several key advantages that compensate for its energetically expensive synthesis: (i) it can retain water, protecting the embedded microbes against drought; (ii) it can entrap and accumulate nutrients from the environment thus allowing survival in oligotrophic surroundings, and (iii) it can shield the bacteria from toxic metals but also antimicrobial compounds [22,23,24].

Exopolysaccharides, the carbohydrate-based component of the EPS, often represent the major portion of the matrix. They can be made up of one or more types of monosaccharides (homo- versus heteropolysaccharides), and can be either neutral or charged. In particular, polycationic or polyanionic (i.e., highly positively or negatively charged, respectively) carbohydrates aid the absorption of water molecules and ions, and the formation of a stable network by acting as a glue between individual cells while also providing a scaffold that allows the stratification of the bacterial community [17,25]. Another key component of microbial biofilms is extracellular DNA (eDNA), which may originate from concerted cell lysis of a small biofilm subpopulation. Its function has remained elusive for many years. However, DNase I treatment of early-stage biofilms causes destabilization and leads to premature dispersion, underlining the structural role of DNA within the 3D matrix. Additional roles have also been proposed, such as a source of nutrition for other colonies, and as a DNA pool for horizontal gene transfer within the biofilm community [26,27].

Finally, the EPS contains extracellular proteins, including both glyco- and lipoproteins. In addition to their roles in scaffolding, cell-to-cell interaction, and adhesion, proteins play important enzymatic functions in various metabolic processes inside the bio-film matrix, and they facilitate the controlled breakdown of the various EPS components and thus enable dynamic biofilm remodeling [28,29]. Among the most prevalent proteinaceous structural components of the biofilm matrix are the functional amyloid fibers called curli, produced by numerous Enterobacteriaceae, and the various curli-like adhesins from other species [30]. These fibrils considerably contribute to the mechanical properties of biofilms. Furthermore, carbohydrate-binding proteins, such as lectins, are frequently found and assumed to provide structural integrity by functioning as adapters between the different EPS components [31,32]. Noteworthily, for each polymer component found in the EPS, there are also enzymes that facilitate their turnover, such as saccharolytic enzymes, proteases, DNases and lipases [22]. This can occur for nutritional purposes, during biofilm remodeling and maturation, or to aid in biofilm dispersion and the release of planktonic bacteria. Furthermore, the same enzymes may play central roles during host colonization and infection, for example by degrading or neutralizing host molecules, allowing the bacteria to evade the immune system and propagate more easily.

This takes us to proteases, which not only have a direct influence on the biofilms but also play crucial roles during host infection. Hence, many of them are regarded as major virulence factors. Moreover, in addition to cleaving proteins in the host’s extracellular matrix to ease host invasion, they frequently target and inactivate host proteases or immunoglobulins from the immune system, as well as components of the complement system, allowing the bacteria to remain undetected for an extended period of time. As a result, these extracellular proteolytic activities are often directly linked to pathologies, thus rendering the associated proteases critical targets for medical treatments [33,34,35]. In the next sections, we will give a brief introduction to proteolytic enzymes, to protein secretion, and present several instances of secreted bacterial proteases that act as key virulence factors that directly impact the course of the infection.

## 2. Proteolytic Enzymes—How They Work and What They Do

**Figure 2:**
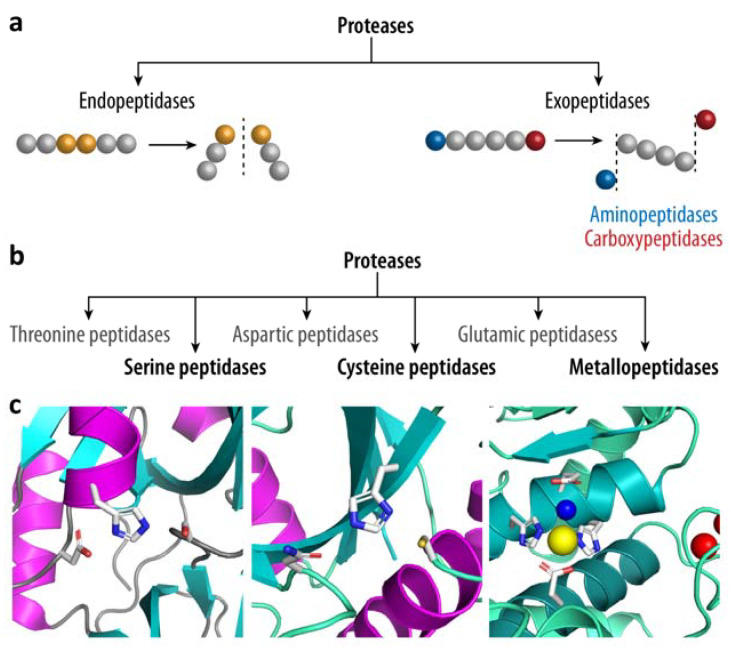
Classification of proteolytic enzymes. (**a**) Proteases can be classified by the site of substrate cleavage. While endopeptidases cleave within proteins, exopeptidases remove N- or C-terminal residues from the target protein. (**b**) Based on the active site responsible for cleavage of the scissile peptide bond, six classes of peptidases can be differentiated, of which threonine, serine and cysteine peptidases act through a covalent acyl-enzyme intermediate, while metallopeptidases and the acidic peptidases utilize an activated water molecule for the nucleophilic attack. (**c**) Active site views of three prominent proteases from *Staphylococcus epidermidis* are shown [40]. Left: the serine peptidase Esp (PDB entry 4jcn [41]), with a catalytic triad (from left to right) formed by Asp159/His117/Ser235. Middle: the cysteine peptidase EcpA with a Asn362/His341/Cys245 triad (based on a AlphaFold2 [42] model of UniProt ID Q5HKF6). Right: the gluzincin metallopeptidase SepA, with His355, Glu375, and His351 as the proteinaceous zinc-binding ligands, and Glu352 as the general base (HE^352^xxH(x)_19_E). The catalytic zinc ion is highlighted in yellow, while two structural calcium ions are shown in red. The depicted SepA active site is based on a AlphaFold2 [42] model of UniProt ID P0C0Q4, while the metal ions and the active site water were inferred from PDB entry 1bqb [43]. All molecular graphics representation were created using an open-source build of PyMOL version 2.5 (The PyMOL Molecular Graphics System, Schrödinger, LLC, New York, NY, USA) [44].

Proteases, also called peptidases, are enzymes that are able to hydrolyze (i.e., break a chemical bond using a water molecule) peptide bonds within proteins and thus break down their substrates into shorter fragments such as peptides, and eventually into amino acids. While exopeptidases use terminal anchoring interactions and thus cleave only the terminal, penultimate, or antepenultimate peptide bond (i.e., removing up to three amino acids from the respective end of the polypeptide chain), endopeptidases target internal peptide bonds by means of so-called context-driven recognition (Figure 2a) [36,37]. In general, proteases utilize the active site water in one of two ways: (i) the protease activates the water molecule (i.e., increasing its nucleophilicity) and thus triggers its attack on the scissile peptide bond (general acid-base catalysis); or, (ii) an activated protein side chain of amino acids such as serine or cysteine acts as the nucleophile, thereby passing through a covalent acyl-enzyme intermediate, which is then resolved by a water molecule to release the bound N-terminal substrate fragment (covalent catalysis). Six distinct classes of proteases have been identified based on the active site and the amino acid or metal ion catalyzing the peptide-bond cleavage, with aspartate, glutamate, and metallopeptidases following acid-base catalysis, while serine, cysteine, and threonine peptidases use covalent catalysis (Figure 2b) [37,38,39].

Metallopeptidases are a large family of peptidases distinguished by an active site metal, typically zinc, that is tetrahedrally coordinated by three proteinaceous ligands and the catalytic water molecule (Figure 2c). Two of the zinc ligands are typically histidines (His; H) derived from a HExxH motif on the so-called active-site helix, which also includes the general acid-base catalyst glutamate (Glu; E). The active site water is sandwiched between the zinc ion and the glutamate, with the latter subtracting a proton and therefore priming the water molecule for the nucleophilic attack on the amide bond carbonyl of the scissile peptide bond upon substrate binding [45,46]. Aspartic and glutamic peptidases belong to the carboxyl peptidase family, which is characterized by a low pH optimum and the respective name-giving acidic active site residue that activates the water molecule. The catalytic dyad of aspartic peptidases is typically formed by a pair of aspartic acid residues, one serving as the general base (i.e., abstracting the proton from the active site water) and the other providing electrophilic support. The situation is similar in glutamic peptidases, where a glutamate residue is typically supported by a glutamine [47,48,49,50].

Serine peptidases represent the most prevalent protease family in bacteria, and they perform so-called covalent peptide hydrolysis. The nucleophilic attack is carried out by a serine side chain inside a catalytic triad, which normally consists of a histidine, aspartate, and serine residue in a highly conserved three-dimensional configuration (Figure 2c). This results in the generation of a covalent acyl-enzyme intermediate, which is then resolved by a water molecule. Every side chain in this acid–base–nucleophile triad has a unique role in catalysis: the acidic aspartate aligns and polarizes the histidine to act as a general base for the serine, enabling it to deprotonate for the nucleophilic attack while also acting as a proton donor for the first cleavage product [51,52]. The process of peptide bond hydrolysis in cysteine peptidases is highly similar, and an equivalent cysteine-histidine-aspartate/asparagine triad is frequently found (Figure 2c). However, because the sulfhydryl group is more acidic than the hydroxyl group of serine, the importance of the aspartate is reduced and thus dyads containing only the nucleophile (cysteine) and base (histidine) are also found [53,54]. Finally, while the overall cleavage mechanism of threonine proteases is similar to serine and cysteine peptidases, a single N-terminal threonine takes over the role of the catalytic dyad. While the secondary alcohol group of the side chain acts as the nucleophile, the N-terminal amino group acts as the general base that either directly or indirectly via a bridged water molecule deprotonates the side chain hydroxyl group to increase its nucleophilicity [55,56].

Beyond doubt, proteases serve critical roles in many, if not all, fundamental biological functions, including protein maturation, signal peptide removal during protein secretion, and as signaling scissors by modulating biochemical pathways [57]. Importantly, more than 95% of the secreted proteases in bacteria are annotated as serine (~48%), metallo (~31%), and cysteine (~17%) peptidases [58], and thus not surprisingly, the proteases involved in biofilm remodeling (e.g., by cleaving EPS proteins or participating in quorum sensing) and pathogenicity (e.g., by cleaving host proteins such as collagen, complement factors, and antibodies), also belong nearly exclusively to these three protease families.

## 3. Protein Secretion to the Extracellular Space

Bacterial proteases can be intracellular, extracellular, or, as in the case of Gram-negative bacteria, periplasmic. While intracellular proteases are commonly associated with protein degradation, they also function as essential signaling scissors, precisely activating and inactivating proteins, thereby altering biochemical pathways and controlling cellular activity [59]. Periplasmic proteases, on the other hand, such as the serine peptidase DegP and the metallopeptidases BepA and YcaL from *E. coli*, are commonly involved in outer membrane protein biogenesis and assembly, and act as quality control factors that clear proteins with folding defects [60]. Finally, the archetypal role of extracellular proteases is likely to scavenge resources, for example by degrading proteins in the surrounding environment and supplying peptides as easily accessible nutrients for the cell. However, they are also involved in biofilm formation and modulation, as already briefly outlined above, as well as in the breakdown of host cell matrices during pathogenesis. Moreover, they have been shown in several instances to increase virulence by targeting components of the host’s immune system, thus allowing bacterial immune evasion, and directly linking secreted proteases to pathogenesis and disease progression [61].

**Figure 3:**
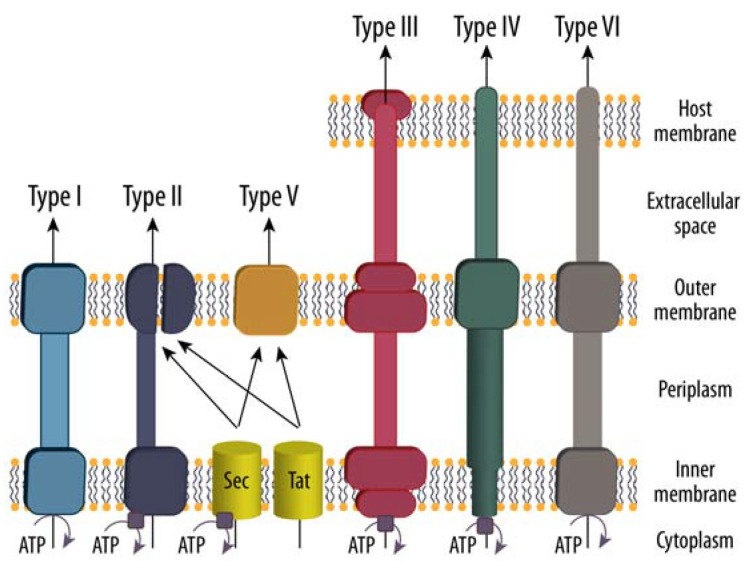
Schematic overview of known secretion systems in Gram-negative bacteria. The basic structural features of the type I (T1SS), type II (T2SS), type V (T5SS), type III (T3SS), type IV (T4SS), and type VI (T6SS) secretion systems are depicted, with the latter three being capable of directly injecting effector proteins into host cells. Sec (general secretion route) and Tat (twin-arginine translocation pathway) transfer unfolded and folded substrates of T2SS and T5SS across the inner membrane, respectively. The other secretion systems are one-step mechanisms that transport their cargo directly from the bacterial cytosol to the extracellular space or into eukaryotic host cells. Importantly, Sec and Tat are sufficient to cross the single bacterial membrane and reach the surface-exposed peptidoglycan layer in Gram-positive bacteria. Figure adapted from [68,69].

Due to the critical roles of extracellular proteins and proteolysis for biofilm formation and bacterial fitness, bacteria have developed several mechanisms for protein secretion. The most commonly used and preserved mechanisms across all kingdoms of life are the “general secretion” (Sec) and “twin arginine translocation” (Tat) pathways (Figure 3) [62]. Both systems rely on signal peptides located at the N-termini of the proteins to be transported. While the Sec pathway primarily conveys unfolded proteins that fold once they reach the periplasm, the Tat pathway transports proteins already folded in the cytosol, distinct signal peptides ensure correct targeting to the respective secretion system [63,64]. Importantly, Sec and Tat both only bridge the cytoplasmic membrane. Thus, they are sufficient for Gram-positive bacteria, where secreted proteins arrive at the peptidoglycan layer and are either covalently linked to the cell wall and thus immobilized, or they passively diffuse to the exterior. Furthermore, some Gram-positive bacteria harness type VII and type VII-like secretion systems (T7SS) to transport proteins across the cell wall. The best studied T7SS are from Mycobacterium tuberculosis, which possesses five sub-types (ESX1 to ESX5), each with distinct secretomes (i.e., set of proteins transported). Importantly, current structural knowledge suggests that the T7SS core complex is positioned solely in the plasma membrane and does not reach the outer mycomembrane, which is located distally to the peptidoglycan layer and represents a unique feature of the mycobacterial envelope. Although specific details on the secretion process are still unavailable, members of the PE-PPE protein family are proposed to form substrate secretion pores in the mycomembrane [65,66,67].

In Gram-negative bacteria, on the other hand, additional components are required to continue protein transport from the periplasm through the outer membrane to the extracellular space, such as the various type II (T2SS) and type V secretion systems (T5SS) (Figure 3). While type V systems are structurally quite simple and are typically made up of a single polypeptide chain that spans the outer membrane and allows for autonomous transport (“autotransporters”), type II secretion systems are a bit more complex since they span both membranes. However, they only have a translocation unit for the outer membrane, and thus, as with the type V system, rely on the prior transport of the cargo proteins into the periplasm by the SEC/TAT pathways. Furthermore, Gram-negative bacteria possess type I secretion systems (T1SS) that directly transport proteins in one step from the cytosol to the extracellular space and highly evolved mechanisms for the direct delivery of bacterial effector proteins and virulence factors into the cytosol of host target cells, such as the type IV and type VI secretion systems, as well as the type III injectosome [62,70].

## 4. The Immune System and How Pathogenic Bacteria Evade to Establish Infection

The immune system is a network of specialized cells and molecules that cooperate to protect the body from infections. Antibodies belong to the immunoglobulin superfamily and represent a vital detection system of the adaptive immune system in vertebrates, capable of detecting and neutralizing foreign substances such as pathogenic bacteria and viruses by binding to surface antigens or secreted effector proteins. Immunoglobulins are composed of two identical heavy chains that designate the antibody class (e.g., IgG, IgM, IgA, IgD, and IgE) and two identical light chains. While the heavy chain alone forms the Fc region, the two antigen-binding Fab regions are each made up of the variable regions of one light and one heavy chain. Communication with the cells of the immune system occurs through the Fc region, which is recognized by surface receptors (FcRs) that are primarily expressed on innate immune cells. Upon the binding of antigen–antibody complexes, the immune cells initiate cell-based and broad-ranging immune responses such as the neutralization and clearance of targeted compounds (e.g., via uptake by macrophages, neutrophils, or natural killer cells) or programming and training of the adaptive immunity (e.g., after internalization by dendritic cells, the presentation of processed antigenic peptides on major histocompatibility complex (MHC) proteins to naïve T cells leads to their expansion and maturation into antigen-specific effector and memory T cell populations) [71,72].

Another pathway initiated by the recognition of the Fc region of an antigen–antibody complex is the classical complement cascade, which is part of the innate immune system and constitutes a powerful barrier against pathogens by mediating bacterial lysis, triggering phagocytosis, and recruiting immune cells [73,74]. When IgM or IgG binds to a bacterial surface antigen, complement complex C1 recognizes the respective Fc region and initiates a series of proteolytic cleavage events that culminate in the formation of C3 convertase (C4bC2b) and the cleavage of C3 into C3a and C3b. While the anaphylatoxin C3a recruits leukocytes, C3b continues the cascade by forming C5 convertase (C4b2b3b) and interacting with additional components to generate the membrane attack complex (C5b-9), which then causes bacterial lysis by forming a pore in the bacterial cell membrane. Alternatively, the lectin pathway can activate the complement system, in which mannose-binding lectin (MBL) takes over pathogen recognition by binding to carbohydrates or glycoproteins on the bacterial surface. MBL oligomers are then recognized by one of three mannose-binding lectin-associated serine proteases (MASPs) in their inactive zymogen form. Binding leads to MASP activation, allowing for the cleavage of C4 and C2, and thus to the formation of C3 convertase and downstream processing as in the classical cascade. Additionally, there is the alternative pathway that involves factors such as B, D, H, and I that are exclusive to this complement pathway. Together with C3b, they produce an alternative C3 convertase (C3bBb) which can activate more C3 and thus enhance the cascade. Notably, while bacterial and fungal cell wall components enhance C3bBb formation, the presence of mammalian cells inhibits it [73,74,75]. Due to the pivotal role of both immunoglobulins and the complement system for host defense, it is not surprising that bacterial proteases capable of targeting these molecules represent major virulence factors that facilitate infection and bacterial survival by disarming the immune system.

Antimicrobial peptides (AMPs) are another effective first line of defense against pathogenic microorganisms by the host innate immune system. They exhibit a typical length of 10 to 50 amino acids and can cause irreversible cell membrane damage. They are characterized as being either α-helical, β-sheet, or extended coil based on their secondary structure, with the latter being disordered on their own. Because of their amphipathic properties (i.e., exposing both hydrophilic and hydrophobic regions), these host defense peptides can interact with bacterial membranes through their hydrophobic interfaces while interacting with polar components via their hydrophilic regions, resulting in a destabilization of the membrane [76]. AMPs comprise a diverse collection of peptides with limited sequence conservation that are primarily stored in the lysosomes of macrophages and polymorphonuclear leukocytes, with cathelicidins and defensins being the most prominent representatives. Importantly, cathelicidins require proteolytic activation; for example, the only cathelicidin described in humans, the α-helical AMP LL-37, requires maturation from its precursor CAP-18 by the serine peptidase proteinase 3. Defensins likewise need proteolytic processing for activity; however, many defensins have been described in man. They are classified as α- and β-defensins in vertebrates based on their amino acid sequence and their synthesis by primarily neutrophils or epithelial cells, respectively. While they share a conserved triple-stranded antiparallel β-sheet structure constrained by intramolecular disulfide bonds, the molecular differences between individual molecules can be significant. However, defensins are always cysteine-rich and cationic at neutral pH, and they exhibit subclass-specific disulfide connectivity (Cys1–Cys6, Cys2–Cys4 and Cys3–Cys5 in α-defensins and Cys1–Cys5, Cys2–Cys4 and Cys3–Cys6 in β-defensins) [76,77,78].

**Figure 4:**
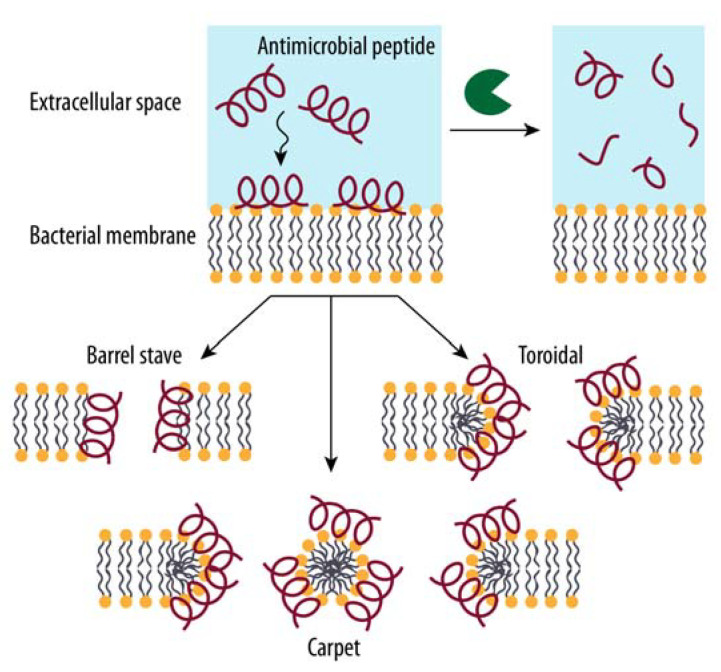
Models of lipid membrane disruption by antimicrobial peptides. Antibacterial peptides are first adsorbed to the membrane surface. Once a certain threshold is reached, membrane disruption occurs. In the barrel-stave model, the peptides are aligned parallel to the phospholipids of the bacterial membrane, generating a continuous barrel-like pore. In the toroidal pore model, a similar hydrophilic transmembrane channel is created, but with the lipid moieties folding inwards, and the hydrophilic phospholipid heads participate in channel formation. In the carpet model, accumulated peptides break down the lipid bilayer in a detergent-like manner into micelle-like structures surrounded by the peptides. Importantly, secreted bacterial proteases, indicated as green Pacman shapes, can cleave and hence inactivate antimicrobial peptides. Figure adapted from [77,79].

Several modes of AMP action have been proposed (Figure 4). In the barrel-stave model, the peptides interact with the membrane via their hydrophobic faces while engaging with each other through their hydrophilic regions, resulting in a perpendicular arrangement of the AMPs to the membrane surface and the formation of a hydrophilic channel. The toroidal model is quite similar, except that lipid molecules intercalate between the individual peptides, and thus the formed channel is created by both the antimicrobial peptides and the hydrophilic head groups of the phospholipids. In the carpet model, however, the peptides bind parallel to the membrane surface and disrupt the membrane in a detergent-like fashion by creating micelle-like units, thus dissolving the cell membrane [77,78].

However, just as vertebrates have developed various defense mechanisms, pathogens have evolved multifaceted strategies to avoid or inactivate both the adaptive and the innate immune system to ensure their survival and spread to different sites within a host, such as by changing their proteins on the surface, or by secreting proteases that can target antibodies, complement factors, or AMPs. For example, by cleaving antibodies between the Fab and Fc regions, the recognition and effector domains of the antibody are separated, inhibiting downstream signaling and thus allowing bacteria to stay undetected, leading to enhanced bacterial survival. Moreover, numerous bacterial proteases have been shown to cleave and neutralize antimicrobial peptides (Figure 4, top right) or to inactivate various components of the complement cascade, hence preventing not only the assembly of the membrane attack complex, but also deterring the recruitment of immune cells to the site of infection [76,80,81].

Among the various proteases capable of cleaving immunoglobulins, the cysteine peptidases IdeS (streptopain) and SpeB (streptococcal erythrogenic toxin B) from the Gram-positive pathogenic bacterium *Streptococcus pyogenes* are the best studied [81]. IdeS is a highly selective protease that separates the Fc and Fab regions of IgG antibodies by sequentially cleaving the two heavy chains of the lower hinge region. While two conserved glycine residues (Gly236-Gly237) form the preferred cleavage site, the Gly-Ala motif observed in IgG2 can also be processed, but with decreased efficiency [82,83]. Importantly, cleavage of just one of the Fc heavy chains already results in an approximately 20-fold reduction in affinity to the corresponding Fc receptor, leading to reduced or eliminated bacterial cell clearance [84]. Unlike IdeS, which has a high degree of specificity, its structural homolog SpeB exhibits a significantly broader substrate profile [85]. In addition to multiple proteins from the host matrix, SpeB is capable of cleaving complement factor C3 as well as immunoglobulins IgG, IgM, IgA, and IgD, but only IgG and IgD cleavage occurs in the hinge region [86]. Furthermore, a recent study emphasized the importance of SpeB during infection, discovering that its specific inhibition resulted in a 28% decrease in bacterial survival in the presence of human neutrophils, confirming the notion that SpeB-mediated proteolysis impairs complement-mediated host defense [87].

Another intriguing set of proteases secreted by Streptococci are the subtilisin-like cell-envelope serine peptidases (CEPs), which are covalently attached to the bacterial cell wall due to their C-terminal LPXTG motif for sortase-mediated processing [88]. While ScpC (streptococcal chemokine protease C; also known as SpyCEP) inactivates interleukin 8 (IL-8) by removing its C-terminal region required for signaling and neutrophil recruitment [89], the C5a peptidase ScpA destroys the chemotactic function of this anaphylatoxin [90]. As a result, both proteases aid the pathogen in evading the host’s immune system, while in the absence of either ScpA or ScpC, significantly increased bacterial clearance is observed as the neutrophil clearance mechanism via so-called neutrophil extracellular traps (NETs) is not impaired by these two proteases [91].

A wide variety of secreted proteases that interfere with the host immune system are also described in Gram-negative bacteria. One of them is the serine peptidase Pic secreted by enteroaggregative *E. coli* (EAEC) strains. Pic shows a broad substrate specificity profile capable of degrading multiple complement system components such as C2, C3, C3b, C4 and C4b, and thereby aiding immune evasion [92]. Enterohemorrhagic *E. coli* (EHEC) produces EspP, a serine peptidase and Pic homolog that likewise inactivates C3/C3b and C5, interfering with the complement cascade and thus preventing bacterial opsonization and complement-mediated killing and phagocytosis [93]. *E. coli* also possesses a β-barrel-shaped outer membrane aspartyl endopeptidase of the omptin protease family named OmpT, a housekeeping protease that degrades foreign peptide material encountered by the bacterium [94]. Unsurprisingly, the same protease is also active against certain antimicrobial peptides (AMPs). This was discovered by comparing the effects of protamine on wild-type versus OmpT knock-out strains, and observing that only wild-type could clear the AMP and protect the cells from damage. On the other hand, deletions in the serine endopeptidase DegP or the metallopeptidase pitrilysin had no effect on protamine susceptibility [95]. However, *E. coli* also possesses other mechanisms for AMP inactivation and clearance.

For example, even though increased OmpT activity correlates with symptomatic uropathogenic *E. coli* (UPEC) infections, the minimum inhibitory concentration of the human antimicrobial peptide LL-37 remained unchanged in a deletion mutant, despite OmpT being capable of partially cleaving LL-37 [96]. However, the most virulent UPEC strains encode not only for OmpT but also for an OmpT-like protease called ArlC. Additionally, in combination, these strains can degrade and inactivate a much wider selection of AMPs [97]. In *Salmonella enterica*, the outer membrane aspartyl peptidase PgtE, which exhibits a 46% sequence identity with *E. coli* OmpT, confers resistance against the antimicrobial peptides C18G and LL-37 [98]. Moreover, plasmid-derived PgtE complementation in Salmonella knock-outs and recombinant expression in *E. coli* demonstrated activity against human complement factors C3b, C4b, and C5, highlighting its capacity to interfere with several lines of host defense. Additionally, PgtE mutants showed reduced growth in the presence of normal human serum but not with heat-inactivated serum, indicating that PgtE confers a certain level of resistance against certain proteinaceous human serum components [99].

In *Pseudomonas aeruginosa*, on the other hand, LL-37 has been shown to be capable of disrupting initial surface attachment and biofilm maturation [100]. However, the secreted metallopeptidase LasB, an elastase, can dampen the impact and improve cell survival by cleaving the AMP at multiple sites [101]. Consequently, even though LasB does not provide complete protection, the inferred increased AMP tolerance may allow for biofilm establishment and development, and is thus crucial for pathogenicity. Furthermore, due to its broad substrate specificity, LasB is also reported to degrade various extracellular matrix proteins, causing severe tissue damage while aiding host invasion [102], and cleaves both complement components [103] and immunoglobulins [104,105], thus facilitating immune evasion.

Another Gram-negative bacterium capable of modulating the immunoglobulin response is *Neisseria meningitidis*, a bacterial colonizer of the human nasopharynx in approximately 10% of human adults. However, when leaving its commensal environment, Neisseria harnesses its IgA1-specific serine peptidase IgA1P, which is also capable of degrading IgG3 [106], and an IdeS-like peptidase capable of cleaving the IgA hinge region [107], to evade the immune system, thereby causing blood sepsis and bacterial meningitis [108].

## 5. Biofilm Remodeling and the Role of Proteases in Virulence

Extracellular proteases execute crucial roles throughout the biofilm life cycle, and they frequently represent virulence factors. Likewise, bacterial biofilm formation and modulation capacities are increasingly recognized as so-called passive virulence factors [109]. Proteolytic activity against proteins of the host extracellular matrix (ECM) often represents a first crucial cornerstone during colonization, as seen for example in clostridial infections and the rapid collagen degradation mediated by their secreted collagenases [110,111,112], or for enteroaggregative *E. coli* (EAEC) and its serine peptidase Pic, which readily cleaves mucins, heavily O-glycosylated linear glycoproteins that are secreted by higher organisms to protect the surfaces of epithelial cells [113]. Moreover, as shown in studies using colonoids (i.e., an organoid derived from human colon crypts), the increased colonization rate of EAEC goes hand in hand with improved biofilm formation capabilities, and both are linked with proteolytic activity [114].

Another major proteolytic virulence factor is fragilysin, a secreted 20 kDa zinc-dependent metallopeptidase that is also known as *Bacteroides fragilis* enterotoxin (BFT), from enterotoxigenic *Bacteroides fragilis* (ETBF). This bacterium is not only linked with acute diarrhea and inflammatory bowel disease, but is also associated with colorectal cancer [115,116]. Fragilysin can cleave a wide variety of ECM substrates, including E cadherin, collagen type IV, actin, fibrinogen, and myosin, thus disrupting the cell-to-cell junctions and disrupting the integrity of the intestinal epithelial barrier and allowing the bacterium to penetrate and invade the host [117,118]. Notably, enterotoxigenic ETBF strains (i.e., strains that express fragilysin) have a greater tendency to form biofilms [119,120], and mouse studies using ETBF strains resulted in mucosal thickening, infiltration of inflammatory cells, crypt abscesses, epithelial cell exfoliation, erosion, and ulcerations in all mouse strains used, and led to even lethal colitis in germfree mice [121]. Furthermore, fragilysin has been associated with colorectal cancer development resulting from the oxidative stress provoked by the upregulation of spermine oxidase due to E-cadherin degradation [122]. 

Another prominent protease capable of cleaving the adherens junction protein E-cadherin is the homooligomeric serine peptidase high temperature requirement A (HtrA) from the group 1 carcinogen *Helicobacter pylori* that colonizes the hostile environment of the stomach [123]. HtrA removes the ectodomain of this tumor suppressor on gastric epithelial cells, thus disrupting the epithelial integrity and propelling the infection. The relationship between HtrA and virulence was further confirmed using a HtrA overexpression system, which resulted in increased (i) rates of E-cadherin cleavage, (ii) bacterial transmigration, and (iii) delivery of the effector protein CagA into host cells [124]. HtrA also plays a key role in bacterial protein quality control, both protease-independent and protease-dependent, by acting as a molecular chaperone for certain nonnative polypeptides while degrading other misfolded proteins, which may explain why its knock-out leads to increased sensitivity to thermal, osmotic, and acidic stress, as well as amplified susceptibility to the amino nucleoside antibiotic puromycin [125,126]. Notably, in *Streptococcus mutans* biofilms, a lack of HtrA results in aberrant biofilm structures with granular patches instead of a smooth confluent layer. This biofilm phenotype may be linked to the role of HtrA in the processing and maturation of various extracellular proteins, such as glucan-binding protein B but also surface-associated glycolytic enzymes such as glucosyltransferases and fructosyltransferase, thereby connecting HtrA activity with biofilm formation and maturation, alongside its role in host invasion [127].

One key group of adhesion proteins involved in *Staphylococcus aureus* biofilm formation is the biofilm-associated protein (Bap) family, which has also been shown to play an important role in adhesion to the surface of epithelial cells and thus in pathogenic biofilm formation [29]. This Bap-dependent biofilm formation process is regulated by several proteases such as the metallopeptidase aureolysin and the staphylococcus serine peptidase A (SspA) [128]. However, the exact molecular mechanism is still unclear, as the various known substrates and the two proteases are strongly interconnected (e.g., aureolysin is required for activation of the SspA zymogen), and as it is likely that additional factors are involved, a phenomenon frequently described as the protease web [129]. In a related study, it was demonstrated that the biofilm matrix interferes with the entry of *S. aureus* into epithelial cells [130], emphasizing the critical role of proteases in biofilm dispersal, for the controlled release of the pathogen from the EPS, and thus for virulence and pathogenicity [131,132] Consequently, aureolysin represents a key virulence factor by cleaving not only proteins from the host’s extracellular matrix and innate immune system, but also of the bacterial cell wall and the biofilm EPS, and by proteolytically activating other secreted toxins. This greatly emphasizes the complex roles of proteases in biofilm biology and bacterial infection on a variety of levels and through a number of different mechanisms that go far beyond cleaving a single substrate.

Furthermore, overexpression of SasG (surface protein G) in *S. mutans* has been found to result in polysacharride-independent biofilm formation, highlighting its role in surface adherence [133]. Sortases likewise play a key role during early surface attachment, especially in Gram-positive bacteria, as they covalently bond adhesion proteins such as the major surface protein P1 to the bacterial cell wall, allowing for protein-mediated cell–cell or cell–surface interactions [134]. In turn, and equally important, protease-mediated adhesin degradation represents a key mechanism during enzyme-driven biofilm dispersal [135], and certain cell-associated proteases may be sortase-dependent. Furthermore, *S. mutans* deletion mutants of sortase A (StrA) were unable to form cell aggregates, likely due to the lower quantity of proteins being anchored to the peptidoglycan surface layer of the cell wall [136], and thus sortases are commonly recognized as critical virulence factors in Gram-positive bacteria [137]. This is further supported by an in vivo nasopharyngeal colonization model in chinchillas, where StrA-positive bacteria were detectable in the nasopharynx even 21 days after application, while sortase mutants were not [138]. Finally, the autolysin-mediated cell lysis of a biofilm subpopulation to provide eDNA for the EPS is also regulated by proteases, as shown in *Staphylococcus epidermidis* biofilms [41,139].

All of these examples demonstrate the intricacy of the numerous mechanisms that drive biofilm development and bacterial pathogenicity, as well as the critical involvement of proteases in these processes, making them not only essential virulence factors but also important therapeutic targets, as they could allow one to inhibit biofilm maturation and dispersal [140]. Furthermore, biofilm-remodeling enzymes may be used to specifically target bacterial biofilms, thereby disrupting biofilm integrity and thus increasing its susceptibility to antibiotics. Additionally, because of reciprocal host–pathogen coevolution, many of these proteolytic virulence factors exhibit high substrate specificity and processivity, rendering them highly attractive for both biomedical and biotechnological applications [141].

## 6. Biomedical and Biotechnological Applications of Bacterial Proteases

Various bacterial proteases have been explored for medicinal purposes, and some have even been approved as therapeutics. Two of the probably best-known protease therapeutics are the botulinum toxins A and B, two metallopeptidases secreted by *Clostridium botulinum* and commercialized as Botox and Myobloc, respectively [142,143]. Notably, despite different substrate specificities, they both cleave components in the synaptic fusion complex, thus abolishing the release of the neurotransmitter acetylcholine into the synaptic cleft. Besides their use for cosmetic purposes, Botox is approved for the treatment of urinary incontinence associated with primary bladder muscle overactivity, abnormal muscle tightness due to prolonged muscle contraction (spasticity), and axillary hyperhidrosis (excessive underarm sweating), among other medical indications [143,144,145], while neck dystonia (severe neck muscle spasms) and chronic sialorrhea (drooling or excessive salivation) may be treated with Myobloc [146]. Furthermore, the large secreted collagenolytic zinc-metallopeptidases ColG and ColH from *Clostridium histolyticum* are used for the treatment of Dupuytren’s contracture and Peyronie’s disease [147]. However, despite good therapeutic performance, the drug was recently pulled from a number of markets including the European Union based on commercial grounds, while still being available in the USA [148]. Recently, IdeS from *Streptococcus pyogenes* (Imlifidase^®^) was used to desensitize patients with strong positive crossmatches for liver transplantation, enabling the procedure [149]. Similarly, the IgA peptidase from *Haemophilus influenzae* was able to deplete the IgA complexes deposited in mouse glomeruli, and thus could represent a treatment option for IgA nephropathy [150].

In addition to these biomedical applications, various bacterial proteases are also used in biotechnology. For example, the glycylglycine endopeptidase lysostaphin from *Staphylococcus simulans* is a potent antimicrobial against *S. aureus*, and its transgenic expression protected cows from bacterial mastitis [151]. Furthermore, treatment of roasted peanut extracts with the *Bacillus licheniformis* serine peptidase subtilisin (Alcalase^®^) has been shown to significantly reduce the IgE reactivity and thus the allergenicity of the derived products, and thus may render common foods containing trace amounts of peanuts from production less dangerous for people with a peanut allergy [152]. Bacterial proteases are also used in the laundry detergent industry. For example, alkaline proteases from *Bacillus* spp. have been used since the 1960s as additives due to their robustness and capacity to remove protein stains [153].

Finally, immunoglobulin-cleaving peptidases from *Streptococcus pyogenes*, such as IdeS and SpeB, are increasingly used in the biopharmaceutical industry to specifically digest antibodies and render them suitable for middle-up proteomic analysis and especially glycoprofiling by mass spectrometry [85,154,155], for example, to compare originator and biosimilar therapeutic monoclonal antibodies. Additionally, since the global market for therapeutic antibodies is predicted to be over USD 300 billion by 2025 [156], demand for these and similar enzymes is expected to surge over the next few years.

All these examples greatly highlight how bacterial virulence factors and proteases involved in biofilm remodeling can be transformed from "foes" during infection and pathogenesis into “friends” for biotechnological and biomedical applications. Surprisingly, this context-dependent potential of beneficial versus detrimental proteolysis is frequently overlooked, while it could enable new research avenues and therapeutic strategies.

## 7. Conclusions

Proteolytic enzymes play intricate roles in many biological systems, including biofilm development and pathogenicity. With this review, we tried to provide a comprehensive reference point for early-career scientists working on microbial proteases and interested in how these enzymes impact bacterial life. We have highlighted several secreted proteolytic enzymes with well-defined roles in biofilm formation, maturation, and dispersion, and showcased proteases that act as virulence factors in host colonization and immune evasion by degrading components of the host extracellular matrix and the immune system. We then conclude with documented biotechnological and biomedical applications of some of secreted bacterial proteases, including their use as laundry detergents, in mass spectrometry, and to desensitize donor organs for patients with strong positive crossmatches.
